# Calmodulin Activation by Calcium Transients in the Postsynaptic Density of Dendritic Spines

**DOI:** 10.1371/journal.pone.0002045

**Published:** 2008-04-30

**Authors:** Daniel X. Keller, Kevin M. Franks, Thomas M. Bartol, Terrence J. Sejnowski

**Affiliations:** 1 The Salk Institute, Computational Neurobiology Laboratory, and Howard Hughes Medical Institute, La Jolla, California, United States of America; 2 Howard Hughes Medical Institute, The Salk Institute, La Jolla, California, United States of America; 3 Division of Biological Sciences, University of California San Diego, La Jolla, California, United States of America; 4 Neurosciences Graduate Program, University of California San Diego, La Jolla, California, United States of America; University of Southern California, United States of America

## Abstract

The entry of calcium into dendritic spines can trigger a sequence of biochemical reactions that begins with the activation of calmodulin (CaM) and ends with long-term changes to synaptic strengths. The degree of activation of CaM can depend on highly local elevations in the concentration of calcium and the duration of transient increases in calcium concentration. Accurate measurement of these local changes in calcium is difficult because the spaces are so small and the numbers of molecules are so low. We have therefore developed a Monte Carlo model of intracellular calcium dynamics within the spine that included calcium binding proteins, calcium transporters and ion channels activated by voltage and glutamate binding. The model reproduced optical recordings using calcium indicator dyes and showed that without the dye the free intracellular calcium concentration transient was much higher than predicted from the fluorescent signal. Excitatory postsynaptic potentials induced large, long-lasting calcium gradients across the postsynaptic density, which activated CaM. When glutamate was released at the synapse 10 ms before an action potential occurred, simulating activity patterns that strengthen hippocampal synapses, the calcium gradient and activation of CaM in the postsynaptic density were much greater than when the order was reversed, a condition that decreases synaptic strengths, suggesting a possible mechanism underlying the induction of long-term changes in synaptic strength. The spatial and temporal mechanisms for selectivity in CaM activation demonstrated here could be used in other signaling pathways.

## Introduction

When calcium ions enter a neuron they can induce a wide range of outcomes in a variety of neuronal signaling pathways, including those responsible for both persistent increases and decreases in synaptic strength [1]. Dendritic spines, the sites of most excitatory synapses in the central nervous system, serve to chemically compartmentalize local changes in calcium ion concentration (Ca^2+^, henceforth referred to as “calcium”) [2], [3], [4]. Synapse-specific changes in synaptic strength depend on activation of multiple calcium-dependent signaling molecules in individual spines. However, it is not clear how selective activation of particular signaling pathways can be achieved within a single spine head less than 1 µm in diameter since calcium in free solution can rapidly diffuse across this distance.

Spines serve as chemical compartments for calcium-driven reactions. Calcium in the spine head is isolated from the rest of the dendrite by the spine neck, where the length and width of the neck defines coupling between the two compartments [5], [6] and can be regulated by neural activity [7]. The spine head itself is not well-mixed, for even within the confines of the simulated compartment, long-lasting calcium gradients can persist for many tens of microseconds. Such microdomains and the concomitant modulation of calcium signals within them have been studied in a variety of contexts. For example, Crank-Nicholson diffusion calculations can predict calcium microdomains at distances below 100 nm in spines [8]. Calmodulin located near calcium channels can direct channel regulation, and indeed the effective local calmodulin level near calcium channels has been show to be in the millimolar range [9]. Microdomain regulation of calcium channels mediated by CaM is lobe-specific [10]. Furthermore, activation of CaM in microdomains is likely to depend upon the diffusion constant of CaM [11]. The amplitude and extent of calcium within the spine also influences spine motility through processes mediated by actin and myosin [12]. Synapse-to-nucleus signaling mediated by extracellular signal-regulated kinase (ERK1/2) is initiated by calcium microdomains at synaptic N-methyl-D-aspartate receptors (NMDARs) [13]. Presynaptically, calcium can become highly elevated for submillisecond intervals [14], activating local sensors [15].

Specific activation of a particular signaling cascade may depend on a sensor that reads the spatio-temporal average of intracellular calcium in the entire spine. Alternatively, spatially distinct sensors may detect highly localized calcium elevations. In dendrites, but not necessarily spines, specific activation of different protein effectors downstream of calcium entry has been shown to depend on the source of calcium entering the cell, including NMDARs [13], [16] and voltage-dependent calcium channels [17], [18]. In hippocampal and neocortical pyramidal cells, the two main sources of spine calcium are voltage-dependent calcium channels and NMDARs. Calcium-permeable α-amino-3-hydroxy-5-methylisoxazole-4-propionic acid receptors (AMPARs), while present at these synapses, make no measurable contribution to the calcium current. [19], [20], [3], [21]. Calcium enters through voltage-dependent calcium channels during backpropagating action potentials, and through synaptic NMDARs when pre-synaptic glutamate release and postsynaptic depolarization are coincident [22], [3], [23]. The precise distribution of voltage-dependent calcium channels within spines remains poorly characterized, but they are generally assumed to distribute evenly over the surface of the spine. NMDARs, in contrast, localize to the postsynaptic density [24], [25]. The different locations of these two calcium sources should produce different spatial calcium profiles in the spine immediately after influx. However, these profiles will only have physiological relevance if they activate different downstream targets.

Calmodulin is a calcium-dependent protein required for the initiation of many different signaling pathways including long-term potentiation (LTP) and long-term depression (LTD) [26]. Calmodulin has two lobes, each of which can be independently activated by binding two calcium ions [27]. Calmodulin has a relatively low calcium affinity, and slow mass-action kinetics [28]. If equilibration of calcium within the spine is extremely rapid, then formation of the four calcium-bound activated CaM should be independent of either its spatial distribution in the spine or the source of calcium entry.

To test the hypothesis that CaM activation is sensitive where it is located within the spine, we developed a biophysically realistic Monte Carlo model of a dendritic spine that included calcium influx, extrusion and buffering. The model, implemented in MCell [29], [30], simulates the diffusion of each calcium ion with a random walk at high temporal and spatial resolution, as well as biochemical reactions between calcium and other molecules in signal transduction cascades. This approach preserves the stochasticity inherent in signaling with a small number of molecules. Our results show that approximations based on the assumption that calcium is well mixed are invalid and that strong calcium gradients can persist long enough to activate CaM in microdomains under conditions that lead to LTP and LTD.

## Materials and Methods

The activation of postsynaptic NMDA receptors by glutamate released in an idealized, 3D neuropil has been described elsewhere [31], [32], [33]. Here we present the methods used to model the voltage-dependent gating of calcium channels and calcium influx, the binding of calcium to various intracellular binding partners, and the establishment of intracellular calcium homeostasis. To model the calcium dynamics in the spine head, the model includes voltage-dependent calcium channels, calcium pumps, NMDA and AMPA receptors, and calcium binding proteins. MCell models the chemical behavior of discrete particles but not electrical properties. To accommodate electrical effects, a NEURON simulation was coupled to the MCell model.

MCell uses rigorously validated and highly optimized Monte Carlo algorithms to simulate the random-walk Brownian motion of discrete diffusing molecules and concomitant uni- and bi-molecular chemical reactions in a complex three dimensional environment reflecting realistic cellular microstructure. Thus, the impact of sub-cellular organization on the temporal and spatial evolution of biochemical reaction-diffusion systems can be studied using MCell. We used version 3.0 of MCell, which differs from previous versions of MCell in that it allows pairwise interactions between diffusing molecules (http://www.mcell.cnl.salk.edu). We were therefore able to model binding of diffusing calcium with diffusing endogenous calcium binding proteins (CBPs) and exogenous fluorescent calcium indicators.

To model a system with MCell it is necessary to specify 1) the geometry of the sub-cellular structures of the system, 2) the diffusion constants and initial locations of diffusing molecules, 3) the locations of transmembraneous or scaffold-tethered effector molecules, 4) the reaction mechanisms and kinetic rate constants governing the interaction of diffusing molecules with each other and effector molecules, and 5) an appropriate time step and number of iterations with which to simulate the spatial and temporal evolution of the system [29], [30]. According to the statistics of a Brownian dynamics random walk as well as Monte Carlo reaction-diffusion algorithms, the average radial distance traveled in a random walk step and the probability that binding occurs both depend on the value of the simulation time step. The ratio of the simulation time step to the mean lifetimes of the chemical reactant states determines the numerical accuracy of MCell simulations [29], [30]. In general, the time step should be chosen so that the probability that binding occurs during the span of one time step is less than 1 (generally not greater than 0.5) to obtain errors of less than 1–2%.

This MCell model differed from previous models of spine microdomains in that discrete molecules were represented in 3D space. In principle, for simulations with a large number of particles, similar results for calcium profiles could result from the numerical solution of partial differential equations (PDEs) in 3D space. We chose to use MCell for two primary reasons. First, MCell explicitly accounts for diffusion and interaction of discrete particles at high spatial resolution with complex cellular geometry at much less computational expense as compared to PDE and other stochastic methods. The geometry of the model shown here represents an intermediate step along the path to simulation of a more realistic geometry reconstructed from electron-microscope tomography. Consideration of complicated geometric constraints would prove difficult to address with other numerical tools currently available. The spatial resolution of MCell depends only on the simulation time step and not on the granularity of a finite element mesh. Second, MCell incorporates stochastic effects such as binding and unbinding of glutamate to discrete channels, the stochastic flickering of activated channels, and binding and unbinding of individual calcium ions with diffusing endogenous proteins and calcium indicator. Stochastic effects are particularly relevant when the number of participating molecules is small. In the relatively large spine used in this model (volume 0.125 fL) an average resting calcium concentration of 50 nM entails only 3–4 free calcium ions. The macroscopic concept of concentration breaks down under such conditions. The relatively small numbers of calcium ions and other signaling molecules means that consideration of discrete particles will become increasingly important for detailed, biologically realistic simulations.

### Geometry of the MCell Model

**Figure 1:**
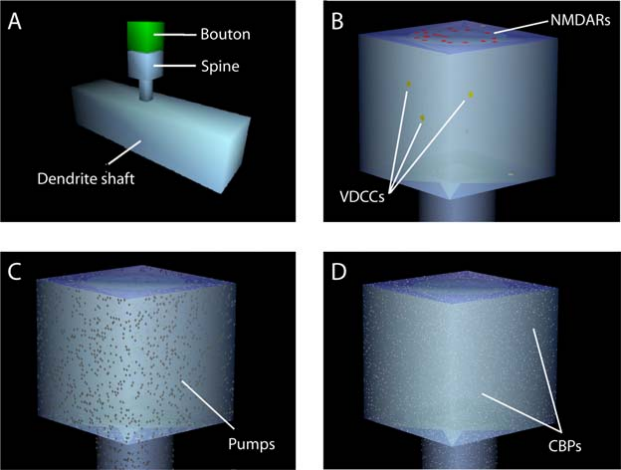
Schematic representation of the model. (A) A segment of dendrite with a single spine (grey). A presynaptic bouton (green) was separated from the synaptic face of the spine by a 20 nm cleft. (B) voltage-dependent calcium channels (yellow) were randomly distributed at low densities across the spine and dendrite membranes, whereas NMDARs (red) and AMPARs (not shown) were restricted to a patch centered on the synaptic face of the spine. (C) Calcium pumps were distributed evenly on the cell membrane. (D) CBPs were uniformly distributed in the interior.

The model encompassed a 4 µm×4 µm×4 µm volume of simplified neuropil composed of cuboidal elements, as previously described [31], [32]. The presynaptic bouton consisted of a cuboid 0.5 µm on a side adjacent to the spine head, creating a 20 nm synaptic cleft (Fig. 1). The spine head, also a 0.5 µm cuboid, was connected to a dendritic shaft by a neck measuring 0.5 µm long and 0.2 µm wide. The dimensions were consistent with average measurements derived from transmission electron microscopy [34]. The model included extrasynaptic elements representing other dendrites, axons, and glial components of the neuropil. For clarity these are not shown in Fig. 1.

### NEURON Simulations and Coupling to MCell

We adapted a previously published NEURON model of a layer 5 neocortical pyramidal cell (Figure S1A) [35] to compute the time series of dendritic voltage and calcium channel gating parameters. This was used to compute a time series of voltage-dependent open probabilities (P_open_) for each channel-type, which in turn was then converted to a time series of kinetic rate constants [29], [30]. MCell then read the files of kinetic rate constants in order to calculate open-closed transitions of voltage-dependent calcium channel and blocked-unblocked transitions of NMDARs.

Action potentials in the NEURON model were induced with a brief somatic current injection (3 nA for 1.5 msec). The action potentials propagated back into the dendritic arbor (Figure S1B).

A pair of connected pyramidal cells has ∼5 synaptic connections, and induction of an action potential in one cell induces an excitatory postsynaptic potential of approximately 2 mV peak amplitude and 30 msec half-width, as measured in the soma of the other [23]. Five synapses were therefore placed on the cell (Figure S1E). We assumed that release at each synapse was reliable and equal in strength. The exact number and strength of concurrent synapses is not a key parameter since these make a minimal contribution to relief of magnesium ion blockade in NMDARs at the synapse of interest. Excitatory postsynaptic potentials were simulated as synaptic current injections in the NEURON model,
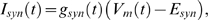
(1)where I*_syn_*(*t*) is the synaptic current and *E_syn_* is the synaptic reversal potential (0 mV). Synaptic conductance, g*_syn_*(*t*), was modeled as an alpha function described by
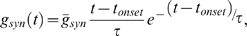
(2)where 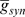 is the maximal synaptic conductance, τ is the decay constant and *t_onset_* is the time of the beginning of the excitatory postsynaptic potential. The spine head simulated in MCell was assumed to be isopotential. An appropriately sized somatic excitatory postsynaptic potential resulted for τ = 2.5 ms and 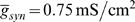. Activation of the proximal apical synapse alone (Figure S1F) resulted in a 5.8 mV voltage increase when measured locally, but only 0.4 mV in the soma.

### Voltage-Dependent Gating of Calcium Entry

Voltage-dependent calcium channels were placed on the dendritic and spine membrane in the MCell simulation. A five-state model for voltage-dependent calcium channels was adopted [36]. The rates governing transitions in this model depend upon the membrane voltage. The NEURON-derived changes in membrane potential were converted into the time-varying rates for each of the transitions and imported into MCell as a time series.

A model of ligand-gating of the L-mode of NMDA receptors [37] was modified to take into account the voltage dependence of the magnesium block. The different ligand-gated states of NMDA receptors were made voltage-dependent, so that each ligand-dependent state could either be blocked or unblocked. State transitions were governed by opening (k_unblock_) and closing (k_block_) rate constants,
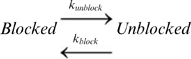
(3)


The probability of a receptor being unblocked was
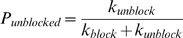
(4)


Assuming a constant extracellular magnesium concentration of 1 mM, the standard relation [38] was used:
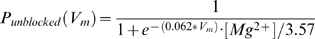
(5)where P_unblocked_(V*_m_*) is the voltage-dependent unblocked probability of a single channel. Each state of the NMDA channel shuttles between blocked and unblocked substates with a blocking rate k_block_ unblocking rate k_unblock_. Taking k_block_ at a fixed rate of 2000 per second, k_unblock_ for each time point was determined by equating equations 4 and 5 and solving for k_unblock_ [39]. The values of k_unblock_ were then imported into MCell as a time series governing the unblocking rate of NMDA channels. The rates for ligand-dependent state transitions were identical whether the channel was in a blocked or an unblocked state. Depolarization associated with the action potential substantially reduced the magnesium block on NMDA.

### Calcium Currents through Open Channels

The large difference between intracellular and extracellular calcium concentrations produces rectifying currents through calcium channels best described by the Goldman-Hodgkin-Katz equation. These currents often have nearly linear I-V relationships at physiological potentials, with different apparent calcium reversal potentials for different channel types [40]. To simulate the passage of ions through an open channel, we therefore assumed ohmic conductances and a constant, non-depleting extracellular calcium concentration. Thus, the rate at which calcium ions entered the cell through a single channel (*r_channel_*) was
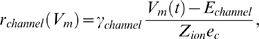
(6)where γ*_channel_* is the single channel conductance, *E_channel_* is the apparent reversal potential, *z* is the valence of the ion (z = 2 for calcium) and *e_c_* is the elementary charge, 1.6×10^−19^ C. Thus, the average number of ions that entered the cell through a single channel per time-step, ΔN*_channel_*, was
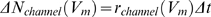
(7)where Δ*t* is the time interval. Ion channel flux is a Poisson process. The probability that exactly *n* ions enter through an open channel, *p*(*n*), on any single time step was therefore given by
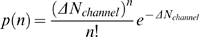
(8)


For each iteration and channel, the value of a single random number was used to choose *n* according to the probability distribution, *p*(*n*). The entry of calcium ions was modeled by generating the *n* ions at the cytoplasmic side of the channel. As voltage changed, r*_channel_* and N*_channel_* were updated accordingly.

The I-V relationship of voltage-dependent calcium channel is linear over the range of voltages in the spine (−65 mV to −3 mV; from NEURON simulations), with an apparent reversal potential of 45 mV [41], [42]. Two inactivating voltage-dependent calcium channels and one non-inactivating voltage-dependent calcium channel have been reported per dendritic patch [43]. Their distinction between inactivating and non-inactivating voltage-dependent calcium channels was based on differing responses to prolonged step depolarizations. Here, the activating depolarization was the brief action potential, and we assumed deactivation-rates far greater than inactivation-rates. Consequently, these channels were grouped together as a single type of voltage-dependent calcium channel. Assuming an average patch size of 1.5 µm^2^ [43], we inserted voltage-dependent calcium channels at a density of 3 per µm^2^, corresponding to, on average, ∼4 voltage-dependent calcium channels in the spine. The voltage-dependent calcium channels were distributed evenly over the surface of the spine except in one simulation examining the effect of clustering them at the postsynaptic density. These values were consistent with published estimates of the number of calcium channels in spines [44]. The single-channel calcium conductance was 2.5 pS [45].

### Glutamate Receptors

NMDA receptors were placed on the postsynaptic membrane within a small circle (350 nm diameter) at a density corresponding to approximately 20 NMDA receptors per synapse (Fig. 1B), consistent with physiological, anatomical, and simulation estimates [46], [47], [25], [32]. Over a range of negative membrane potentials, approximately 10% of the current through NMDA channels was carried by calcium which, in the absence of the magnesium block, has an approximately linear I-V-relationship through the range of physiological voltages [48], [49], [50], [51]. We therefore modeled calcium entry through NMDA channels according to Eqn. 1 with the calcium conductance g*_NMDA_* set to 4.5 pS, or 10% of the 45 pS total conductance [47]. At physiological calcium concentrations, *E_NMDA_* extrapolated to 3 mV [52]. The blocking and unblocking of the channels was governed by Eqn. 5. Calcium-impermeable AMPA receptors (830 per µm^2^) were included for completeness [31].

### Calcium Leak and Extrusion

Calcium was extruded from the cytoplasm by pumps distributed across membrane surfaces (Fig. 1C). Calcium pumps were modeled with Michaelis-Menten kinetics according to
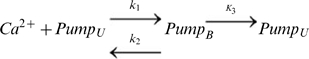
(9)where *k*_3_ is the turnover rate at which a calcium-bound pump returns to the unbound state with the removal from the simulation of a calcium ion. The maximum pumping velocity (V*_max_*) is the product of the turnover rate and the pump density and was defined per unit area in the model. Calcium extruders fell into two classes: Plasma membrane calcium ATPases (PMCA) and sodium calcium exchangers (NCX). They were placed on the surface of the cell membrane at a density of 150 per µm^2^ PMCA and 32 per µm^2^ NCX (Fig. 1C), balanced such that each removes approximately the same amount of calcium in response to an action potential. PMCA pumps have a K*_M_* of 0.2 µM [53] and a turnover rate of 100 per second while NCX pumps have a K*_M_* of 3 µM but a turnover rate 10 times higher than that of the ATP-driven pumps [54], or 1,000 per second.

Resting calcium levels were maintained by balancing extrusion with a nonspecific leak current associated with each calcium extruder. Fixing the rate of each leak channel to 25 ions per second per PMCA and 48 ions per second per NCX resulted in a basal dendritic calcium concentration ([Ca^2+^]*_i_*) of 50 nM [5], [55], [56], [57]. Detailed descriptions of the leak and extrusion mechanisms are described elsewhere [32].

### Intracellular Calcium Binding and Calcium Fluorescence

Mobile endogenous calcium binding proteins (CBPs) may be dialyzed during whole-cell recordings [8]. This may explain the low values of endogenous calcium buffer capacity measured using fluorescent calcium transients in spines [3]. Calcium buffering was therefore modeled using two scenarios. When simulating calcium imaging experiments that use fluorescent indicators, we included exogenous Oregon Green BAPTA-1 (OGB1) with specified kinetics and mobility (Table 1) but no mobile endogenous buffer. For simulations of unperturbed neurons 45 µM of mobile calbindin-D28k was included [8]. The calbindin-D28k model had four independent calcium binding sites, two of medium affinity and two of high affinity (Table 1). A basal level of 5 µM of a CBP with fast kinetics was included in all simulations (Table 1). The parameters used are intended to represent a typical neuron, though in all likelihood the buffering capacity of different pyramidal cells in the cortex varies from cell to cell.

**Table 1:** Parameters used for Ca^2+^ diffusion and binding.

Parameter	Value	Source
Calcium diffusion constant	220 µm^2^s^−1^	Adapted from [58]
**Calbindin**		
Medium affinity site association-rate	4.35×10^7^ M^−1^ s^−1^	[59]
Medium affinity site dissociation-rate	35.8 s^−1^	ibid
High affinity site association-rate	0.55×10^7^M^−1^ s^−1^	
High affinity site dissociation-rate	2.6 s^−1^	
Diffusion constant	28 µm^2^s^−1^	ibid
**Fast Calcium-Binding Protein**		
association-rate	6×10^7^ µm^2^s^−1^	
dissociation-rate	1200 s^−1^	
Diffusion constant	0 µm^2^s^−1^	
**Oregon Green BAPTA-1**		[60]
Diffusion-rate	84 µm^2^s^−1^	
Indicator association-rate	0.43×10^9^	
Indicator dissociation-rate	79 s^−1^	
**Calmodulin**		
CaM concentration in the postsynaptic density	260 µM	
C-lobe first association rate	6.8×10^6^ M^−1^ s^−1^	[61], Fit from [28]
C-lobe first dissociation rate	68 s^−1^	ibid
C-lobe second association rate	6.8×10^6^ M^−1^ s^−1^	ibid
C-lobe second dissociation rate	10 s^−1^	ibid
N-lobe first association rate	108×10^6^ M^−1^ s^−1^	ibid
N-lobe first dissociation rate	4150 s^−1^	ibid
N-lobe second association rate	108×10^6^ M^−1^ s^−1^	ibid
N-lobe second dissociation rate	800 s^−1^	ibid

The model also included calmodulin. In the CaM model used, each lobe of CaM binds calcium independently (Figure S2) [61], [62]. Rate constants were determined by fitting the macroscopic rates of calcium association with calmodulin to the model (Table 1). The double-bound form of CaM can modulate calcium binding proteins under certain conditions [63], thereby influencing plasticity.

In the MCell simulations, 750 molecules of CaM were included within the postsynaptic density. In simulations testing the location effects of CaM activation, an additional 750 molecules were distributed uniformly throughout the spine head. 750 molecules in the spine head corresponded to a concentration of 10 µM, while the same number of molecules in the postsynaptic density corresponds to an effective concentration of 500 µM. Though the exact quantity and localization of CaM remains poorly-characterized experimentally, the qualitative differences in activation between postsynaptic density and uniformly-distributed CaM should hold through a wide range of concentrations.

In some simulations, calcium buffers with OGB1-like kinetics were also included. Fluorescence, F(t), was measured as
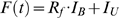
(10)where I*_B_* and I*_U_* are the number of indicator molecules that are calcium-bound and calcium-free, respectively, and R*_f_* is the difference in fluorescence intensity of the calcium-bound versus the calcium-free species of the indicator; (R*_f_* = 9; [64]). Fluorescent transients, (ΔF/F), were measured as
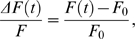
(11)where *F*_0_ is the fluorescence averaged over a pre-stimulus interval. The intracellular concentration of free calcium ions ([Ca^2+^]*_i_*) was measured by counting the number of free calcium ions in a compartment and dividing by its volume. The fluorescence-predicted calcium concentration ([Ca^2+^]*_pred_*), however, was calculated using the standard equation for determining concentration from fluorescence,
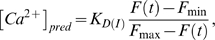
(12)where K*_D_*_(*I*)_ is the dissociation constant of the indicator, F*_min_* equals I*_Total_* (i.e. I*_B_*+I*_U_*) the total number of indicator molecules and F*_max_* equals the product of I*_Total_* and *R_f_* [65].

Fluorescence-predicted [Ca^2+^]*_i_* allows the measurement of buffering capacities [57], [66]. The change in [Ca^2+^]*_pred_* in a compartment immediately after a brief calcium current, such as an action potential, can be described by
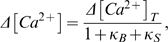
(13)where [Ca^2+^]*_T_* is the total amount of calcium entering the compartment and κ_B_ and κ_s_ are the added and endogenous calcium-binding ratios, respectively. κ_B_ was defined as
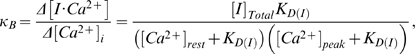
(14)where [*Ca* ^2+^]*_rest_* and [*Ca* ^2+^]*_peak_* are resting and peak values of [Ca^2+^]*_pred_* and Δ[*I ?Ca* ^2+^] is the increase in the amount of calcium-bound indicator. K_D(I)_ is the dissociation constant of the indicator. κ_S_ can then be predicted when plotting the inverse of the change in predicted calcium concentration (1/Δ[Ca^2+^]) against κ_B_ [57], which is the ratio of calcium ions that enter a compartment that are bound by endogenous CBPs to those that remain free. A central assumption in the determination of κ_s_ is that extrusion is very slow compared to buffering. For determination of κ_s_ we therefore measured the change in [Ca^2+^]*_pred_* with different concentrations of OGB1 by running simulations in which the pump and leak densities were set to zero.

Parameters were either selected or adjusted to simulate experiments conducted between 30–32°C. All MCell simulations were run with a time-step (Δ*t*) of 100 ns. Because NEURON simulations were run with an integration time step of 25 µs, all MCell values imported from NEURON were updated only when they changed. The parameter values specified above represent the mean values used. At initialization of each simulation, the number and positions of receptors, channels, pumps and binding proteins were randomly assigned on specified surfaces [30]. Simulations were run on a cluster of 2.2 GHz AMD Opteron workstations running Debian Linux. It took approximately 60 hours of computer time on a single processor to simulate 1 second of real time.

### Calibration of Simulated Action Potentials and Excitatory Postsynaptic Potentials

**Figure 2:**
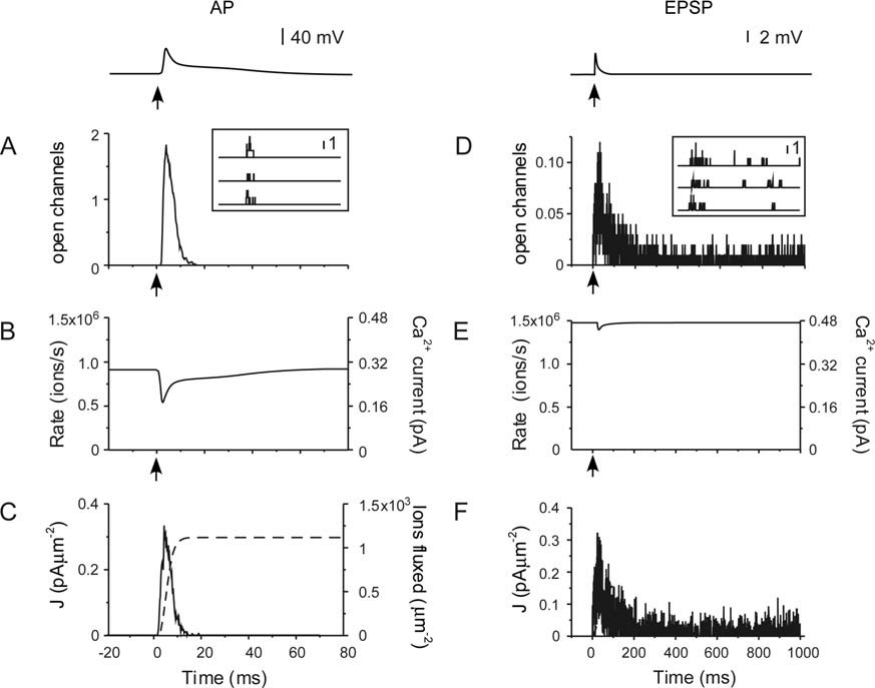
Calcium currents through voltage-dependent calcium channels. Relevant voltage traces at the synapse are shown above each column. (A) Ensemble average (n = 40) of open voltage-dependent calcium channels across 1 µm^2^ of membrane in response to a backpropagating action potential elicited by a current injection into the soma (at arrow). Inset: Single trials show voltage-dependent calcium channel stochasticity. (B) Average voltage-dependent rate of calcium ion flux through a single open voltage-dependent calcium channel during action potential, as derived from the NEURON simulation. (C) Current density (solid trace) and cumulative flux of calcium ions across 1 µm^2^ during an action potential (dashed trace). (D) Ensemble average (n = 100) of open NMDARs across 1 µm^2^ of membrane in response to the release of glutamate (at arrow) and a small depolarization. Inset: Probability distribution of n for a 4.5 pS NMDAR at −65 mV. (E) Average rate of calcium ion flux through a single NMDAR as a function of voltage during an excitatory postsynaptic potential (F) Calcium current density from 10 NMDA receptors distributed across the postsynaptic density (solid trace).

During an action potential, the change in membrane voltage caused a significant decrease in the calcium driving force through a voltage-dependent calcium channel, The number of open voltage-dependent calcium channels in the spine head during an action potential peaked at 1.8±0.75 (n = 20), with the ensemble average number of open channels also reaching a maximum at 1.8 (Fig. 2B). The average flux of calcium ions through voltage-dependent calcium channels peaked at 0.33 pA per µm^2^ with a total flux of 1,276 ions per µm^2^ following an action potential (Fig. 2C).

Following the release of 1,500 glutamate molecules to stimulate an excitatory postsynaptic potential, the peak number of unblocked open NMDARs [31] was 4.5±0.6 channels, with an ensemble average of only ∼0.1 open channels at peak (Fig. 2D). The glutamate number and NMDARs quantities used were calibrated to yield reasonable stimulation characteristics while maintaining fidelity to experimental measurements. The relatively small excitatory postsynaptic potential barely affected the rate of calcium flux through open NMDARs (Fig. 2E). Release of glutamate resulted in a peak calcium current of 0.3 pA µm^−2^ through NMDARs (Fig. 2F). In order to decrease the voltage-dependent magnesium blockade during an excitatory postsynaptic potential, in some simulations an action potential (AP) was applied 10 ms after onset of the excitatory postsynaptic potential (EPSP). Although the total amount of glutamate-bound NMDARs remained the same, the ensemble average number of peak open channels increased to 0.96 during the EPSP-AP stimulation. This conferred a peak synaptic calcium current of 0.18 pA and a total integrated influx of 4,000 ions 1 sec after transmitter release.

## Results

### Simulated Action Potentials and Excitatory Postsynaptic Potentials

**Figure 3:**
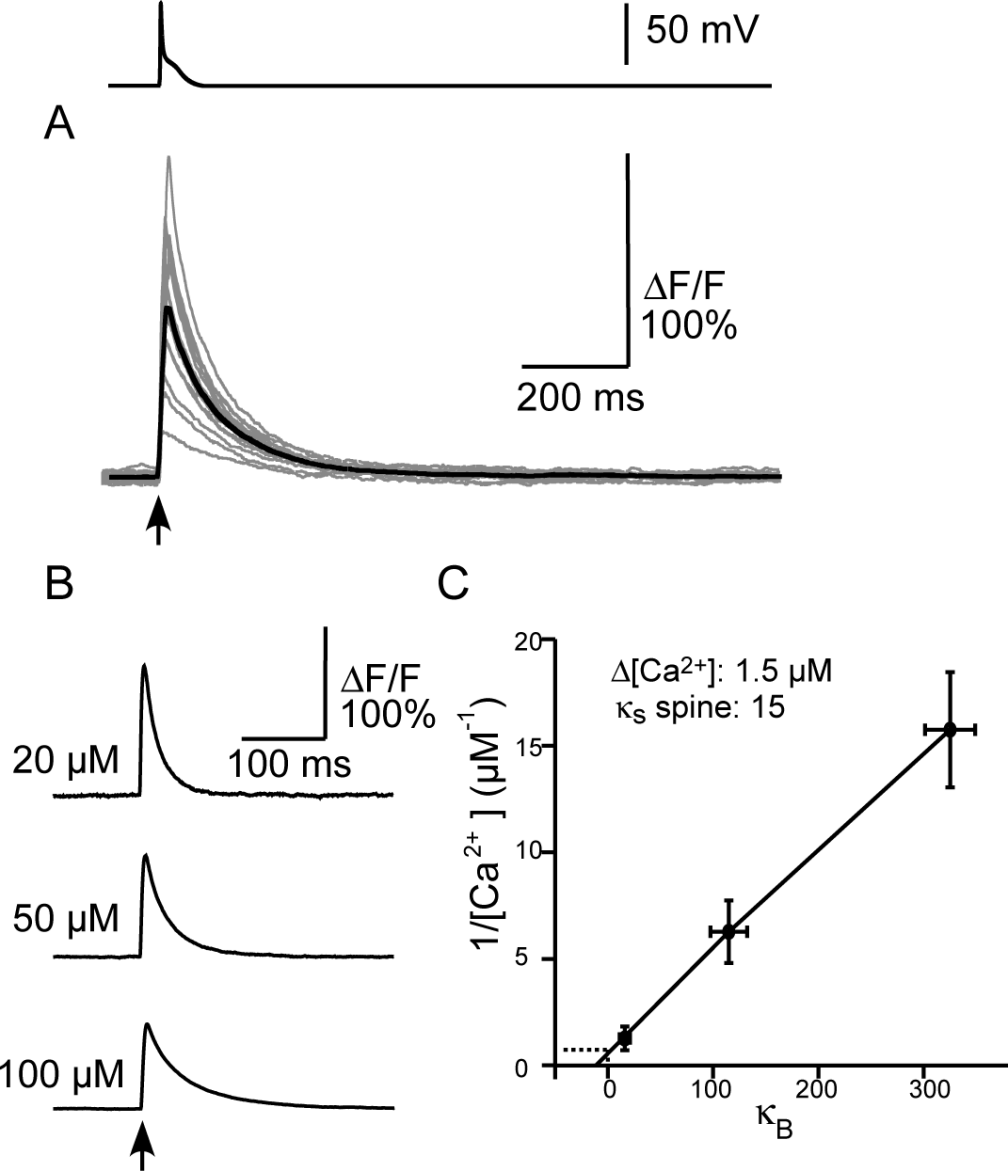
Calcium dynamics following an action potential. (A) Volume-averaged OGB1 (100 µM) fluorescent transients. Grey traces, single trials; black trace, ensemble average of 20 trials. The inset shows the dendritic voltage during the event. (B) Action potential-evoked transients simulated with different concentrations of OGB1. (C) Inverse amplitude of [Ca^2+^]_pred_ as a function of κ_B_. Each point shows the mean±SD for 20 trials with different concentrations of indicator. A regression line (solid line) was fit to the data. Extrapolating the line, the y-intercept gave the estimated amplitude of the unperturbed transient, the x-intercept gives the estimated κ_S_.

To predict the dynamics of calcium at microsecond and nanometer resolutions, the model must first match measurements taken at experimentally observable scales. We therefore began by directly comparing the performance of our model with experimental measures of calcium influx, buffering, and extrusion in simulations of the fluorescence of OGB1 (100 µM) following an action potential (Fig. 3). These simulations were performed in the absence of calbindin-D28k, which is assumed to have dialyzed out of the cell during whole-cell recording. The peak amplitude of the volume-averaged simulated fluorescent transients varied considerably (mean ΔF/F, 0.76; coefficient of variation, 0.27, Fig. 3A) due to the small number of stochastically fluctuating voltage-dependent calcium channels in the spine. The transients had a rapid onset (20–80% rise, 1.4 ms) and a double exponential decay (decay constants, 47.9±9.25 ms and 450±30 ms). These results agree with experimental measures of calcium dynamics in dendritic spines measured with similar concentrations of high affinity indicator [19], [20], [5], [67].

The calcium binding ratio (κ_S_, [54]) provides a quantitative description of the intracellular buffering of calcium ions. Derivation of κ_S_ exploits the dependence of the peak and decay of fluorescent calcium transients on the concentration of exogenous buffer (κ_B_). Our model reproduced this dependence on indicator concentration. The fluorescent transients became smaller and slower with increasing concentrations of OGB1 (Fig. 3B). Plotting 1/[Ca^2+^]*_pred_* versus κ_B_ predicts a peak calcium amplitude with zero added buffer of 1.5 µM and a κ_S_ value of 15 (Fig. 3C). The model was again consistent with experimental measures of calcium influx, buffering, and extrusion following back-propagating action potentials.

NMDARs, situated on the synaptic face of the spine, were the only active calcium source during a subthreshold excitatory postsynaptic potential. Since they are mostly blocked by magnesium at subthreshold voltages, the amount of calcium entering the spine through NMDA receptors during an EPSP was relatively small. Quantal release of glutamate resulted in highly variable fluorescent transients in the spine (Figure S3). Individual traces showed large fluorescence spikes that decayed rapidly after individual NMDARs opened and calcium entered the spine. These rapid calcium transients were not well synchronized, they are not well represented by averaging across trials, which instead convolves the rapid individual transients with the slow gating of NMDA receptors (20–80% rise time, 9.0 ms; decay constant, 254 ms).

### Calcium Indicators Do Not Track Fast Calcium Changes

**Figure 4:**
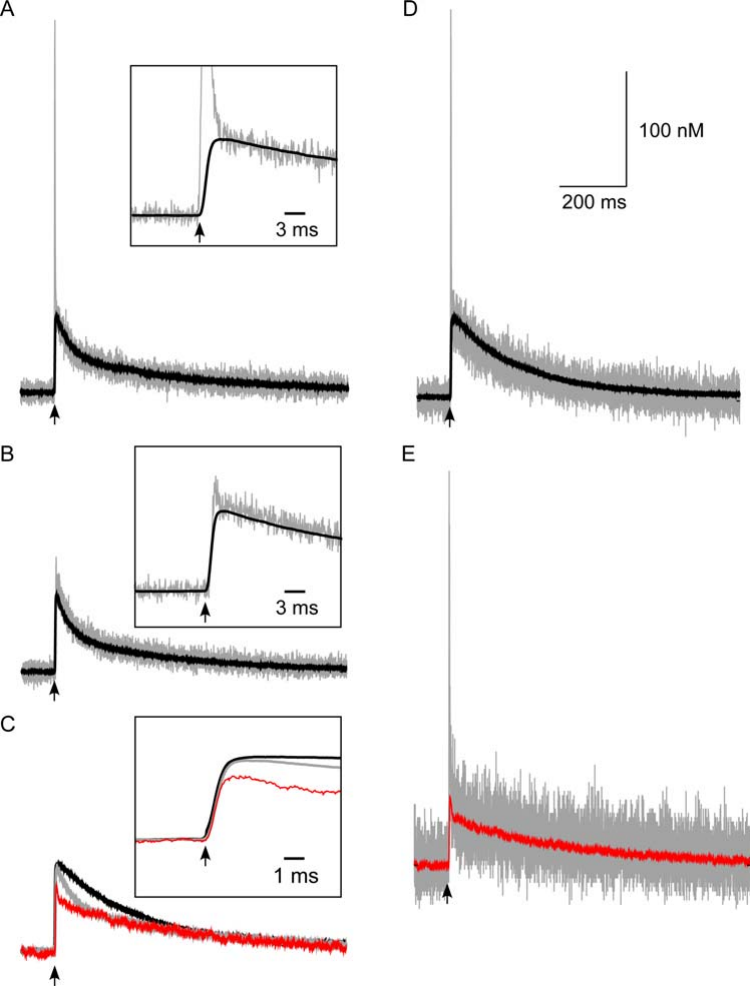
Action potential-induced transients visualized with an indicator. (A) True calcium concentration (grey trace) in action potential simulations run with 100 µM of OGB1. [Ca^2+^]_pred_ (black trace) is derived from the fluorescent transient. The calcium concentration predicted by the dye did not rigorously follow the time course of the actual calcium transient. The inset shows an expanded view of the initial rise segment. (B) Increasing both the on-rate and the off-rate by a factor of 10, thereby maintaining the same K_d_, diminishes the discrepancy between the calcium signal (grey) and the indicator-predicted signal (black). The inset shows an expanded view of the initial rise segment. (C) Immobile indicator caused the indictor calcium signal to linger (black trace) as compared with the signal obtained using indicator that diffused at the normal diffusion-rate (grey trace). Increasing the diffusion constant of the indicator by a factor of 10 (red trace) increased the decay rate from the spine head as well as equilibration with the dendritic shaft. Eventually the calcium signal in all traces converges to the slower dendritic removal rate. The inset shows the first few milliseconds of the event. (D) The predicted calcium (black trace) and actual calcium using an immobile indicator. (E) The predicted calcium (red trace) and actual calcium using a 10x mobile indicator.

How accurately does the OGB1 fluorescence transient predict the underlying calcium dynamics in dendritic spines? On the whole, OGB1 (100 µM) fluorescence accurately reported the volume-averaged calcium concentration in the spine (Fig. 4A). In particular, [Ca^2+^]*_i_* and [Ca^2+^]*_pred_* were indistinguishable at baseline and a short time after the action potential. However, the model illustrates a dramatic difference between [Ca^2+^]*_i_* and [Ca^2+^]*_pred_* immediately after voltage-dependent calcium channels open following an action potential (Fig. 4Ainset). When voltage-dependent calcium channels open, calcium enters the spine and is subsequently bound by OGB1, leading to an increase in ΔF/F. The large difference between the between [Ca^2+^]*_i_* and [Ca^2+^]*_pred_* during the action potential reflects the free cytoplasmic calcium that has not yet bound to OGB1. We explored this interaction in detail, as described below.

Although OGB-1 is based on BAPTA, which is considered a ‘fast calcium buffer’, we wondered whether increasing the speed at which the buffer can bind calcium would increase accuracy in predicting intracellular calcium levels. Increasing both the on- and off-rates of OGB1 10-fold - thereby maintaining the same K_d_ – dramatically increased the accuracy of [Ca^2+^], as reflected by the closer agreement between [Ca^2+^]*_i_* and [Ca^2+^]*_pred_* (Fig 4B). Although the peak value of [Ca^2+^]*_pred_* did increase marginally (by 2 nM), the agreement reflects more effective buffering of calcium rather than better temporal resolution of [Ca^2+^]*_pred_*. We also suspected that [Ca^2+^]*_pred_* might be sensitive to the OGB1 mobility. We therefore compared calcium dynamics with the diffusion coefficient of OGB1 (D_OGB1_) either increased 10-fold or set to zero (Fig. 4C).

The mobility affected how quickly the indicator-predicted transients decayed. When the mobility of the indicator was increased tenfold (Fig. 4C, red trace) the rate of decay of the predicted calcium increased substantially. At longer time intervals the signal tracked the slower decay kinetics of the parent dendrite. With increased mobility, the peak of predicted calcium was slightly less than the control case since some of the bound indicator diffused out of the spine head between the onset of stimulation and the peak of the transient. When the indicator was immobilized (Fig. 4C, black trace), the decay time of the predicted transient increased because bound indicator did not diffuse from the spine.

We systematically varied the dissociation constant and on-rate of the indicator (not shown). For all K_d_ ranges tested, the kinetics of the indicator affected the magnitude of the predicted signal. Faster indicator on-rates allowed better prediction of the actual calcium transient resulting from physiological stimuli. Furthermore, as expected, simulations performed with lower affinity indicator predicted larger free calcium ranges that approached the unperturbed condition.

We also simulated native conditions in unpatched cells having no OGB1 indicator but containing 45 µM calbindin-D28k. Under these conditions, [Ca^2+^]_I_ peaks at 1.7 µM after an action potential, markedly higher than in simulations conducted in the presence of 100 µM OGB1 but without the calbindin-D28k (0.36 µM). After the initial influx of calcium following an action potential, calcium levels dropped precipitously with fast and slow decay time constants of 3 ms and 95 ms. The fast phase of the decay corresponded to rapid buffering of free calcium immediately after closing of the voltage-dependent calcium channels and the slow decay was a function of extrusion as well as the affinity of the CBPs.

In order to understand the extreme case of no endogenous buffering proteins, we also performed simulations in the absence of calbindin-D28k and calcium indicator. Under these conditions, the average action potential induced calcium level over the entire spine head reached a maximum of 11.1 µM. This is significantly higher than the peak of 1.5 µM obtained by extrapolating the predicted calcium obtained at varying levels of calcium indicators to the case of no added indicator, and is a consequence of the inability of indicators to exactly track calcium influx during the action potential.

Calcium transients following synaptic stimulation also resulted in differences between the indicator-predicted calcium transients and the actual calcium traces (Fig. 3B). For simulations with 100 µM OGB1 but without calbindin-D28k, the mean peak [Ca^2+^]*_i_* (i.e. the average amplitude of all peaks, independent of when they occur) was 1.07±0.7 µM but the peak of the averaged calcium transient was only 0.17 µM (Figure S3B). Although [Ca^2+^]*_pred_* more closely matched the temporal properties of the actual free calcium during an excitatory postsynaptic potential than an action potential, it still underestimated [Ca^2+^]*_i_*. The indicator did not properly track the high frequency components of the calcium signal arising from the flickering of NMDA transients during the excitatory postsynaptic potential and also diverged at longer time scales.

What underlies the difference between actual and predicted calcium concentrations? We considered a simplified, well-mixed system without pumps and other calcium sources. The time constant with which the system approaches equilibrium in response to an impulse injection of calcium can be calculated by solving a differential equation. In a more complicated system such as the dendritic spine, with extrusion pumps and diffusion pathways out of the spine head, the system would approach a steady-state rather than an equilibrium. The simple unicompartmental model was represented by the following differential equation for the bound indicator:
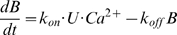
(15)


The concentration of bound indicator B, unbound indicator U, and free calcium in the above equation were set to the appropriate equilibrium values at t = 0. In the above equation k_on_ and k_off_ were the indicator on and off rates. k_on_ was 4.5 ⋅10^8^ M^−1^s^−1^ and k_off_ was 79 s^−1^. In the case of 100 µM total indicator, the unbound indicator concentration was 80 µM under basal conditions with 50 nM free calcium. If free calcium was instantaneously increased by 30 µM the time constant to approach the new equilibrium was 33 µs.

To corroborate the above estimate we simulated (using MCell version 3.0) an isolated cube measuring 0.5 µM on a side containing 100 µM of indicator. If calcium was released uniformly throughout the cube, the MCell result agreed well with the unicompartmental differential equation result (Figure S4A).

However, in real spine heads calcium enters through ion channels in the membrane. Thus, the well-mixed assumption is not a realistic view of calcium dynamics in real cells. If the calcium was released from a point source at the edge of the cube, as if entering through a single ion channel, equilibration was slower (70 µs; Figure S4A).

Additionally, calcium influx during a backpropagating action potential occurs continuously for a period on the order of a millisecond, rather than an instantaneous increase. In this case, the calcium can be expressed as:
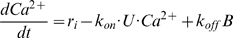
(16)where *r_i_* is the influx rate. We solved the unicompartmental equation of the simplified, well mixed system with an injected calcium influx rate of *r_i_* = 30 mM/s, for 1 ms. At the end of the pulse there was a more pronounced divergence in the indicator-predicted calcium concentration from the actual free calcium concentration (Figure S4B). This occurred because the calcium did not bind the indicator instantaneously and steadily accumulated while calcium was being injected (Figure S4B). Eventually the net rate of indicator binding approached the rate of influx as free calcium levels increased (Figure S4C). When the injection pulse ended after 1 ms, the system equilibrated with the same fast kinetics observed previously for a single instantaneous release. If MCell was used to simulate release of calcium from the center of a one side of a cube, the free calcium level was higher than predicted by the unicompartmental simulation.

This effect is consistent with our observations in MCell that increasing OGB1 binding kinetics increases the accuracy of calcium concentration prediction. This manipulation decreased the time needed for the net binding rate of the indicator to approach the injection rate upon the onset of a pulse, so less excess calcium accumulated.

Because calcium channels and NMDA receptors can open for long periods, during these events the actual free calcium concentration will diverge from the instantaneous concentration predicted by the indicator. Furthermore, the presence of calcium pumps, additional buffers, and diffusion through the spine neck in real spines imposes additional low-pass filtering characteristics upon the system.

### Action Potentials and Excitatory Postsynaptic Potentials Exhibit Different Calcium Spatial Profiles

NMDA channels were clustered at the postsynaptic density. Voltage-dependent calcium channels, on the other hand, through which most of the calcium entered during a backpropagating action potential, were distributed evenly over the surface of the spine. The difference in the placement of calcium sources led to differences in the volumetric profile of calcium in response to both types of stimuli.

**Figure 5:**
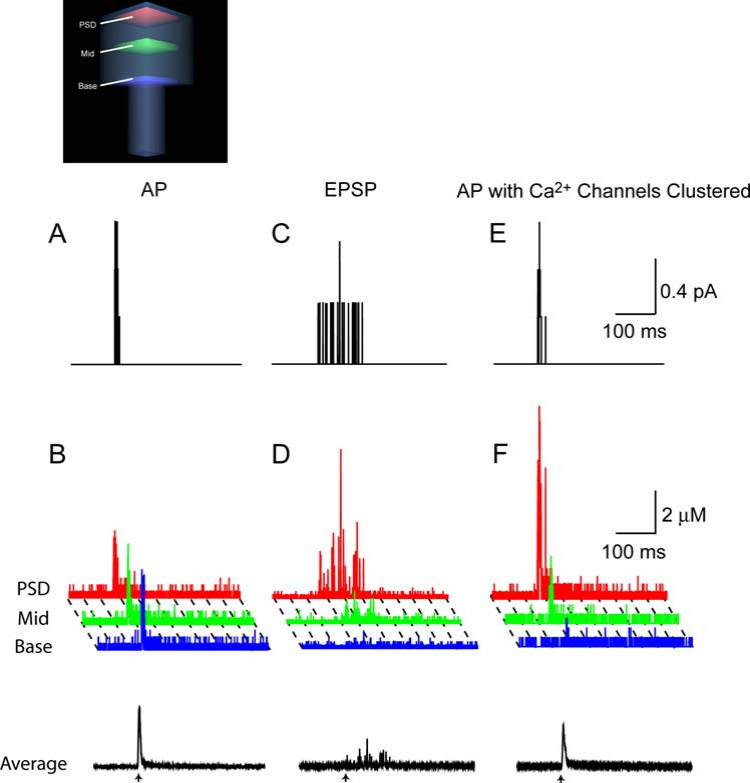
Input-dependent calcium gradients across the spine, in the presence of 45 µM calbindin-D28k. (A) Instantaneous calcium current through voltage-dependent calcium channels in the spine during an action potential. The time of somatic current injections is indicated by the arrow. (B) Schematic (lower left) shows the spine subdivided into three distinct sampling regions: the postsynaptic density (red), the middle (green), and the base (blue) of the spine calcium concentration in each of the three sampling regions is shown during the action potential. The colors of traces correspond to the three sampling regions shown in the schematic. Action potentials did not result in calcium gradients across the spine. The black trace at the bottom of the figure shows the volume-averaged [Ca^2+^]_i_ in the entire spine. (C) Instantaneous calcium current through NMDARs during an excitatory postsynaptic potential. (D) Calcium concentration in each of the three subregions of the spine during an excitatory postsynaptic potential as well as the volume average. Excitatory postsynaptic potentials resulted in large calcium gradients across the spine. (E) Open voltage-dependent calcium channel channels during an action potential simulation in which voltage-dependent calcium channels were clustered at the postsynaptic density. (F) Input-dependent calcium gradients across the spine during the action potential. Calcium concentration is shown in each of the three sampling regions, and the volume average is depicted at the bottom.

The calcium concentration was sampled in three distinct regions of the spine: at the postsynaptic density, in the middle, and at the base of the spine (Fig. 5). We assumed native conditions for these and subsequent experiments: no OGB1 is present and a calbindin-D28k concentration of 45 µM was used.

Backpropagating action potentials, by virtue of the presumably uniform distribution of voltage-dependent calcium channels throughout the spine, generated a relatively uniform calcium distribution. The calcium current underlying the action potential-generated calcium influx occurred over in the course of a few milliseconds (Fig. 5A). The single-trial calcium concentration in the entire spine following an action potential exhibited a sharp peak (Fig 5B, black trace). Since the calcium entered throughout the spine, calcium gradients during an action potential did not occur. Instead, [Ca^2+^]*_i_* in the spine was elevated nearly uniformly (Fig. 5B).

In contrast to the action potential case, the only calcium influx during excitatory postsynaptic potentials occurred through NMDARs restricted to the synaptic surface of the spine. During a single subthreshold excitatory postsynaptic potential, the volume averaged [Ca^2+^]*_i_* (Fig. 5D) and instantaneous NMDAR calcium current (Fig. 5C) in the entire spine were comparable in amplitude to that of the action potential. However when calcium levels were compared across sub-regions of the spine, a large long-lasting calcium gradient extended across the spine during an excitatory postsynaptic potential. Specifically, calcium concentration in the postsynaptic density, where the NMDARs are located, was extremely high but decreased to near resting levels at the base of the spine.

The results were of course predicated on the assumption that voltage-dependent calcium channels were distributed uniformly over the spine head. If they clustered, for example, at the postsynaptic density, a very different action potential calcium profile would result. Figure 5F shows the action potential calcium response in the postsynaptic density, middle, and base of the spine that would occur if all of the calcium channels were to reside at the postsynaptic density. In this case, calcium levels became elevated in the zone directly adjacent to the site of the channels. The high sensitivity to calcium channel placement warrants further studies of voltage-dependent calcium channel localization.

**Figure 6:**
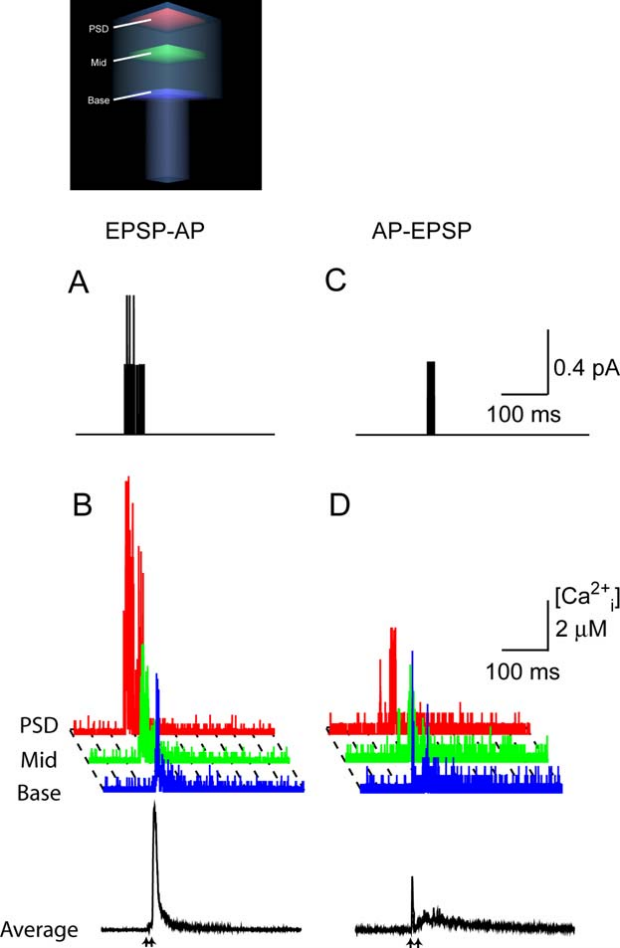
The effect of shifting the order of excitatory postsynaptic potential and action potential stimuli on calcium gradients across the spine, in the presence of 45 µM calbindin-D28k. An excitatory postsynaptic potential (EPSP) preceding an action potential (AP) by 10 ms results in increased calcium influx. (A) Instantaneous calcium current through NMDARs. (B) Calcium concentration in each of the three subregions of the spine during the EPSP-AP event. Note the large calcium gradient across the spine. The black trace shows the volume averaged [Ca^2+^]_i_ in the entire spine when an excitatory postsynaptic potential preceded an action potential by 10 ms. The arrow indicates the time of glutamate release. (C) Open NMDARs when the stimulus is an action potential preceding an excitatory postsynaptic potential by 10 ms. (D) Input-dependent calcium gradients across the spine. The black trace shows the volume averaged [Ca^2+^]_i_ in the entire spine.

In addition to an excitatory postsynaptic potential launched from baseline voltages, we simulated an additional case with the magnesium block relieved by physiological stimuli. In this simulation, a backpropagating action potential (AP) arrived at the spine 10 ms after the presynaptic release of glutamate. The concomitant depolarization abolished the magnesium blockade and permitted much more calcium influx into the spine (Fig. 6A). The supra-threshold EPSP-AP stimulus exhibited higher levels of calcium than did the subthreshold excitatory postsynaptic potential but, as with the subthreshold case, elevated amounts of calcium persisted near the point of entry (Fig. 6B). Reversing the order of excitatory postsynaptic potential and action potential stimuli such that the action potential preceded the excitatory postsynaptic potential did not alleviate the magnesium blockade as much (Fig. 6C). The excitatory postsynaptic potential component of this stimulus did induce a gradient in the spine, but on average the calcium response to an AP-EPSP was much less than that derived from an EPSP-AP (Fig. 6D).

### The Spatial Extent of Microdomains Depends on the Amount of Endogenous CBPs Present

There are conflicting experimental results measuring levels of endogenous calcium buffering [8], [3]. Two possible reasons might account for these conflicting results. Perhaps some CBPs diffuse out of the cell and into the pipette during the indicator equilibration period, thereby attenuating the levels of CBPs. Alternatively, some subsets of pyramidal cells might not express high levels of CBPs. To accommodate both of these possibilities we also simulated excitatory postsynaptic potential responses both with and without 45 µM of calbindin-D28k. Persistent calcium microdomains were observed in excitatory postsynaptic potential simulations both with and without calbindin-D28k (not shown). Simulations without calbindin-D28k manifested larger calcium transients and the calcium gradient induced by an excitatory postsynaptic potential extended farther. Immobilizing calbindin-D28k did not significantly change the spatial extent of the observed microdomains.

### Calmodulin Activates Differently in Response to Action Potentials and Excitatory Postsynaptic Potentials

**Figure 7:**
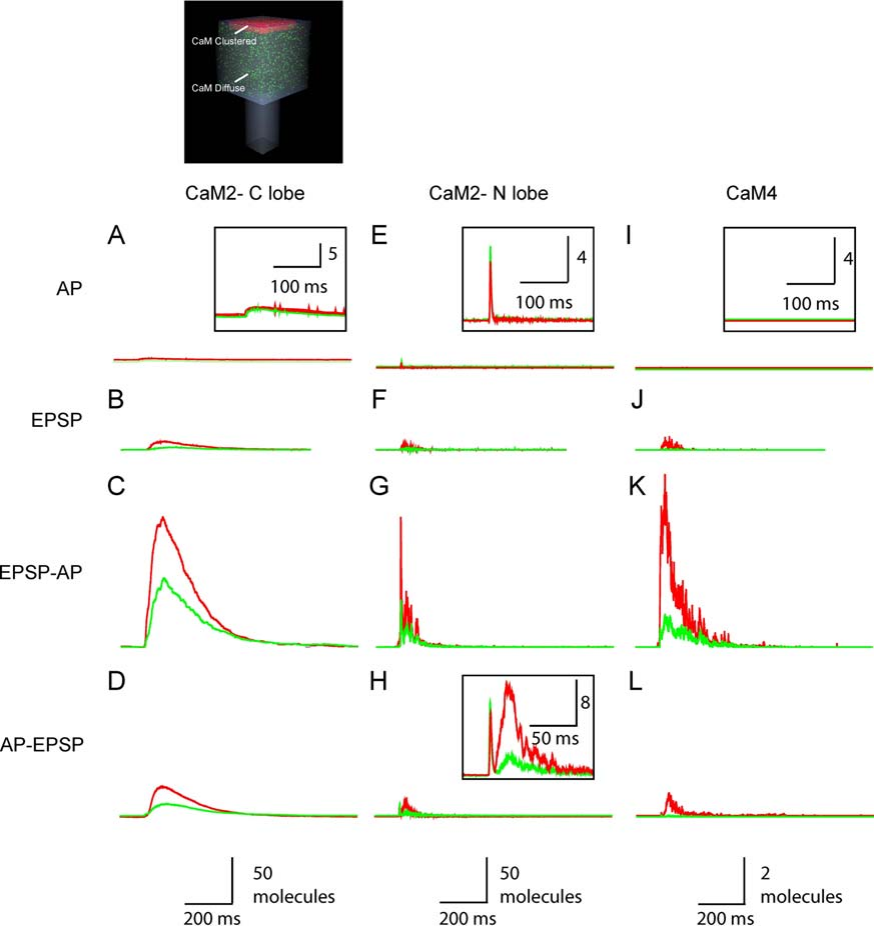
Activation of CaM is sensitive to its distribution and the mode of calcium entry. The schematic shows the two populations of CaM, concentrated in the postsynaptic density and also uniformly distributed through the spine. (A) Action potential (AP)-evoked changes in CaM-2 C-lobe activation with CaM either clustered at the postsynaptic density (red) or uniformly distributed throughout the spine (green). The inset zoom shows no significant difference between clustered and uniform C-lobe responses. (B) Excitatory postsynaptic potential (EPSP)-evoked CaM-2 C-lobe transients. (C) EPSP-AP-evoked CaM-2 C-lobe transients. (D) AP-EPSP-evoked CaM-2 C-lobe transients (E) AP-evoked changes in CaM-2 N-lobe activation. The inset shows a close-up view. The N-lobe of diffuse calmodulin is slightly more activated than that of calmodulin clustered at the postsynaptic density. (F) EPSP-evoked CaM-2 N-lobe transients. (G) EPSP-AP-evoked CaM-2 N lobe transients. (H) AP-EPSP-evoked CaM-2 N lobe transients. The inset shows a close-up view. The N-lobe of diffuse calmodulin was slightly more activated than that of calmodulin clustered at the postsynaptic density in response to the action potential component, but in response to the excitatory postsynaptic potential component the N-lobe clustered calmodulin exhibited a greater response. (I) Action potential induced changes in CaM-4 activation. (J) EPSP-evoked CaM-4 activation. (K) EPSP-AP-evoked CaM-4 activation. (L) AP-EPSP-evoked CaM-4 activation. Each trace is the average of 20 trials.

Are the calcium gradients that occur across the spine following synaptic stimulation physiologically relevant? To examine this question we asked whether CaM responded to the very rapid spikes in calcium concentration seen at the postsynaptic density following influx through flickering NMDARs. Calmodulin is a ubiquitous calcium effector protein with slow mass action kinetics. We monitored the activation of two populations of CaM in the spine. One population of CaM was concentrated at the postsynaptic density and the other was uniformly distributed throughout the spine (Fig. 7upper left illustration). The total number of CaM molecules was equal for the two populations and the distribution of CaM within the spine had no effect on intracellular calcium dynamics following an action potential.

The activated states of calmodulin responded differently depending on the location of the calmodulin and the type of stimulus. Clustering of calmodulin at the synapses versus distributing it uniformly throughout the spine did not significantly affect the amount of double-bound C-lobe CaM that resulted from an action potential (Fig. 7A). However, the uniformly-distributed CaM population exhibited a slightly higher peak in the N-lobe response following an action potential than did the postsynaptic density-clustered CaM population (Fig. 7E). The N-lobe of CaM, with its faster kinetics, was particularly sensitive to the large, local and rapid calcium spikes produced by action potentials. In contrast to the action potential stimulus case, in response to either an excitatory postsynaptic potential or an EPSP-AP stimulus, postsynaptic density-clustered CaM exhibited higher levels of all activated states of CaM (Fig 7 B,C,F,G,J,K, red traces) as compared to the population of uniform CaM (green traces). Even the slower C-lobe exhibited differential activation, with enhanced levels of activation in the postsynaptic density. Thus even molecules with relatively slow mass-action kinetics can respond differentially to gradients within the spine. When an action potential was presented 10 ms before an excitatory postsynaptic potential, an intermediate calmodulin profile combining aspects of single action potential and single excitatory postsynaptic potential responses resulted (Fig. 7 D, H, L). The influx of calcium during the excitatory postsynaptic potential component of the AP-EPSP stimulus was slightly larger than that resulting from a subthreshold excitatory postsynaptic potential alone due to partial magnesium blockage relief afforded by the voltage tail of the preceding action potential.

## Discussion

The Monte Carlo model of intracellular calcium dynamics in spines typical of CA1 pyramidal cell dendrites, which incorporated biophysically-realistic mechanisms of calcium influx, extrusion and buffering and constrained by measured parameters, agreed with experimental measurements of postsynaptic calcium dynamics using calcium-sensitive indicators following both an action potential and an excitatory postsynaptic potential. After matching measurements at larger spatial scales, the model was then used to investigate calcium dynamics at higher spatial and temporal resolution. Activation of CaM depended on the mode of calcium entry and its spatial distribution in the spine. The concentration of calcium-binding proteins such as calbindin-D28k also influenced the extent of spatial microdomains.

Distributing the CaM either clustered in the postsynaptic density or diffusely in the spine head did not significantly affect activation of its C-lobe following an action potential stimulus. The N-lobe activation, however, was enhanced when it was uniformly distributed. The enhanced activation of the N-lobe of the uniform CaM arose because more CaM resided in close proximity to all of the calcium channels spread throughout the spine. Synaptic clustering of CaM in the postsynaptic density greatly increased formation of activated CaM states following an excitatory postsynaptic potential, as compared with diffusely distributing the CaM. Both the C-lobe of CaM and the fully-occupied CaM4 states of CaM have relatively slow kinetics of formation. Thus, calcium gradients in spines persisted for sufficiently long to differentially activate *slow* downstream targets. Enzyme cascades reliant on recruitment of free CaM are therefore expected to be sensitive to the location of the enzymes that bind CaM as well as the diffusion of CaM itself.

Calmodulin increases its affinity for calcium when associated with the proteins it modulates [68]. Therefore, one would expect the actual amount of calcium-bound calmodulin to be greater for stimuli influencing calmodulin in complex with other proteins. The amount of calcium-bound calmodulin would thus be greater than that observed in these simulations, although the qualitative effect should be similar.

It is generally believed that the intermediate levels of calcium give rise to depotentiation or depression, whereas more concentrated calcium pulses cause potentiation. Models of downstream cascades that incorporate calcium dynamics can predict the strength of plasticity in response to different stimuli [69]. The spatial localization of calcium microdomains and the duration of the calcium gradients add an additional layer of complexity since they may serve to differentiate depotentiation and depression processes from those leading to potentiation.

The postsynaptic density, a thicket of proteins situated directly at the site of calcium influx through glutamate-gated receptors, exhibits a high degree of molecular organization [70], [71], [72]. If the equilibration of calcium within a single compartment were sufficiently rapid it would preclude any specificity imparted by spatial localization of different calcium sensors. However, as demonstrated here, the calcium microenvironment experienced within and nearby the postsynaptic density differs markedly from the rest of the spine and can selectively activate signaling complexes. This is consistent with the high degree of organization of proteins comprising the postsynaptic density.

Enzymes residing in the postsynaptic density that are involved in processes leading to potentiation and AMPA clustering, such as CaMKII, would be subject to high effective concentrations of calcium in response to synaptic stimulation. Pathways that lead to depotentiation might involve components that are more uniformly distributed, such as calcineurin or protein phosphatase I. These molecules could interact with the components in the postsynaptic density if they remained activated long enough to diffuse to regions where they could mediate the process of depotentiation. The location, diffusion constants, and kinetics of the relevant molecules remain poorly-characterized but are of key interest in predicting their interactions with calcium in the spine.

At short time scales, beyond the range of most optical measurements, there is a divergence of indicator-predicted calcium concentration from the actual concentration. Prolonged transients occur because calcium accumulates at the onset of the transient before the net indicator binding rate can approach the influx rate. The difference persists throughout the injection. For very fast transients below the steady-state time constant, the on-rate for the indicator may not be fast enough and also binding of calcium to the indicator may be diffusion limited. The last point is salient, for even the ideal indicator with a high on-rate and high K_d_ could consistently under-predict calcium levels due to physical limitations on the maximal rate of encounter between an indicator molecule and a calcium ion. This limit is approached when the reaction on-rate is on the order of 10^9^–10^10^ M^−1^s^−1^. In the simulations we carried out, the diffusion limit did not substantially contribute to under-prediction. Increasing the on-rate and off-rate of the indicator while maintaining the same K_d_ permitted better tracking of the actual free calcium, indicating that the primary cause of divergence was the low on-rate of the indicator. Available calcium indicators seldom have faster on-rates than the values of the on-rates used in these simulations. Furthermore, real indicators make a tradeoff between the contradictory properties of high on-rate and low affinity (high K_d_). It follows that endogenous CBPs transducing calcium signals in spines are subject to similar kinetic constraints.

There are other possible mechanisms that will be explored in future simulations. Spines contain several NMDA receptor subunit types in addition to the NR2A subunit assumed in the model. The model also assumed uniform distribution of voltage-dependent calcium channels based upon a common kinetic model. Recent evidence, however, suggests that there are several types of voltage-dependent calcium channels within the spine and that the admixture of channel types may differ from that found on the parent dendritic shaft [73]. Furthermore, extrusion mechanisms may slow in response to repeated stimuli via a calcium-mediated process [74]. Variability within the signaling cascade imposes additional limitations on the fidelity of the signal relayed by calcium [75].

We conclude that calcium gradients in dendritic spines are sufficiently persistent to differentially activate downstream calcium effector proteins. Thus, functional calcium microdomains in spines are a simple mechanism for imparting selectivity to calcium-dependent signal transduction cascades leading to plasticity and remodeling of dendritic spines and could serve a similar role in other calcium signaling systems such as those in presynaptic terminals and cardiac myocytes.

## Supporting Information

Figure S1 NEURON simulations for voltage-dependent channel gating. (A) Membrane potential measured in a dendritic spine located on the proximal part of the apical dendrite. Action potentials were evoked by short current injections into the soma. (B) An action potential initiated in the soma (blue trace) propagated back into the dendrite (red trace). (C) P_open_ of voltage-dependent calcium channel calcium channels in the spine during an action potential. (D) The gating parameter K_on_ of the NMDA channel during an action potential. (E) Excitatory postsynaptic potentials were simulated by placing 5 synapses at different locations in the dendritic arbor. (F) Five excitatory postsynaptic potentials caused a small deflection in voltage at the soma but a large local depolarization in the spine. (G) P_open_ of voltage-dependent calcium channels in the spine during an excitatory postsynaptic potential. (H) The gating of the NMDA channel during an excitatory postsynaptic potential. The Mg^2+^ block of the NMDA channel was transiently relieved during an action potential but not an excitatory postsynaptic potential.
(0.47 MB TIF)

Figure S2 Calmodulin state diagram. Each lobe of calmodulin can bind 2 calcium ions independently of the other lobe. The N lobe has faster kinetics than the C lobe.
(0.16 MB TIF)

Figure S3 Calcium dynamics following an excitatory postsynaptic potential. (A) Volume-averaged OGB1 fluorescent transients measured in the spine. Thin grey traces are single trials and thick black traces represent the average of 20 trials. (B) Calcium concentration in the spine. The grey trace shows results with 100 µM of OGB1. Also shown is the [Ca^2+^]_pred_ derived from the fluorescent transient (red). A simulation conducted without OGB1 (black trace) also included 45 µM calbindin-D28k, as would be present in unperturbed neurons. Excitatory postsynaptic potentials were elicited by release of glutamate at times indicated by the arrows.
(0.23 MB TIF)

Figure S4 Simulation of 100 µM OGB-1. (A) The predicted (dashed black) and MCell (red) bound OGB-1 closely matched if calcium was uniformly released. If calcium was released from the center of one of the faces in the MCell simulation, it took longer for the system to reach equilibrium (solid black). (B) For a 1 ms duration injection pulse modeled using differential equations, the actual calcium (dashed black) exceeded the indicator-predicted calcium (green). After the pulse was shut off the system quickly equilibrated. If calcium was injected uniformly (red) in an MCell simulation, the results agreed with the differential equation model. However, if calcium was released from the center of one of the faces (solid black) the divergence from the well-mixed unicompartmental model was even greater. (C) The difference between the injected calcium rate and the net rate of binding to the indicator. When channels first opened it took time for the net binding rate to the indicator to approach the influx rate, and during this interval the free calcium increased. This surfeit of free calcium was maintained as long as calcium was injected. The inset shows a magnified difference in rates during the injection. When the injection pulse was shut off the indicator quickly equilibrated with the excess free calcium.
(0.28 MB TIF)
