# Supplementary figures and images for: Calmodulin Activation by Calcium Transients in the Postsynaptic Density of Dendritic Spines
Source: PLoS One. 2008 Apr 30;3(4):e2045. doi: 10.1371/journal.pone.0002045 (PMC2312328; doi:10.1371/journal.pone.0002045)

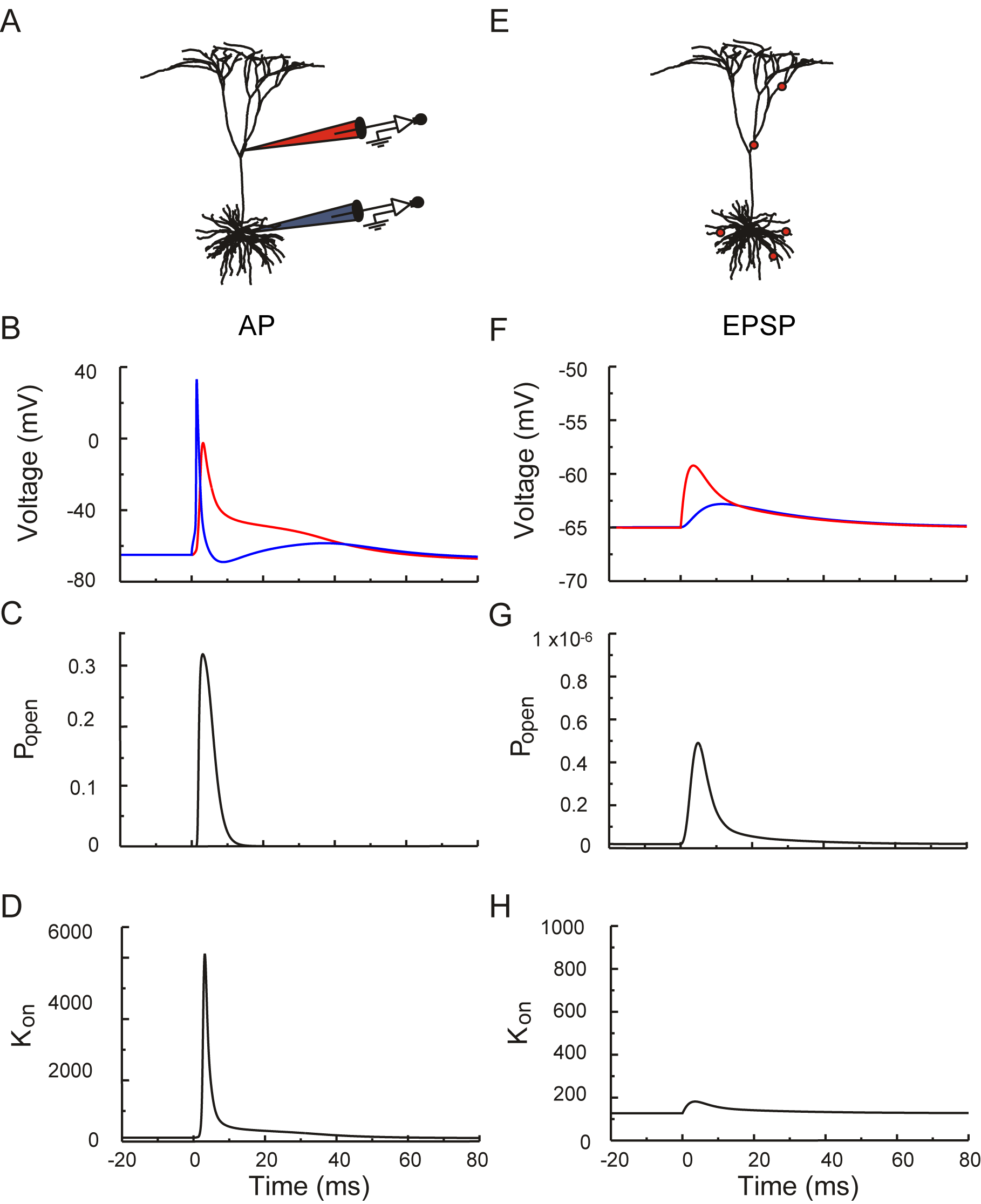

Supplement: Figure S1 — NEURON simulations for voltage-dependent channel gating. (A) Membrane potential measured in a dendritic spine located on the proximal part of the apical dendrite. Action potentials were evoked by short current injections into the soma. (B) An action potential initiated in the soma (blue trace) propagated back into the dendrite (red trace). (C) Popen of voltage-dependent calcium channel calcium channels in the spine during an action potential. (D) The gating parameter Kon of the NMDA channel during an action potential. (E) Excitatory postsynaptic potentials were simulated by placing 5 synapses at different locations in the dendritic arbor. (F) Five excitatory postsynaptic potentials caused a small deflection in voltage at the soma but a large local depolarization in the spine. (G) Popen of voltage-dependent calcium channels in the spine during an excitatory postsynaptic potential. (H) The gating of the NMDA channel during an excitatory postsynaptic potential. The Mg2+ block of the NMDA channel was transiently relieved during an action potential but not an excitatory postsynaptic potential. (0.47 MB TIF) [file pone.0002045.s001.tif]

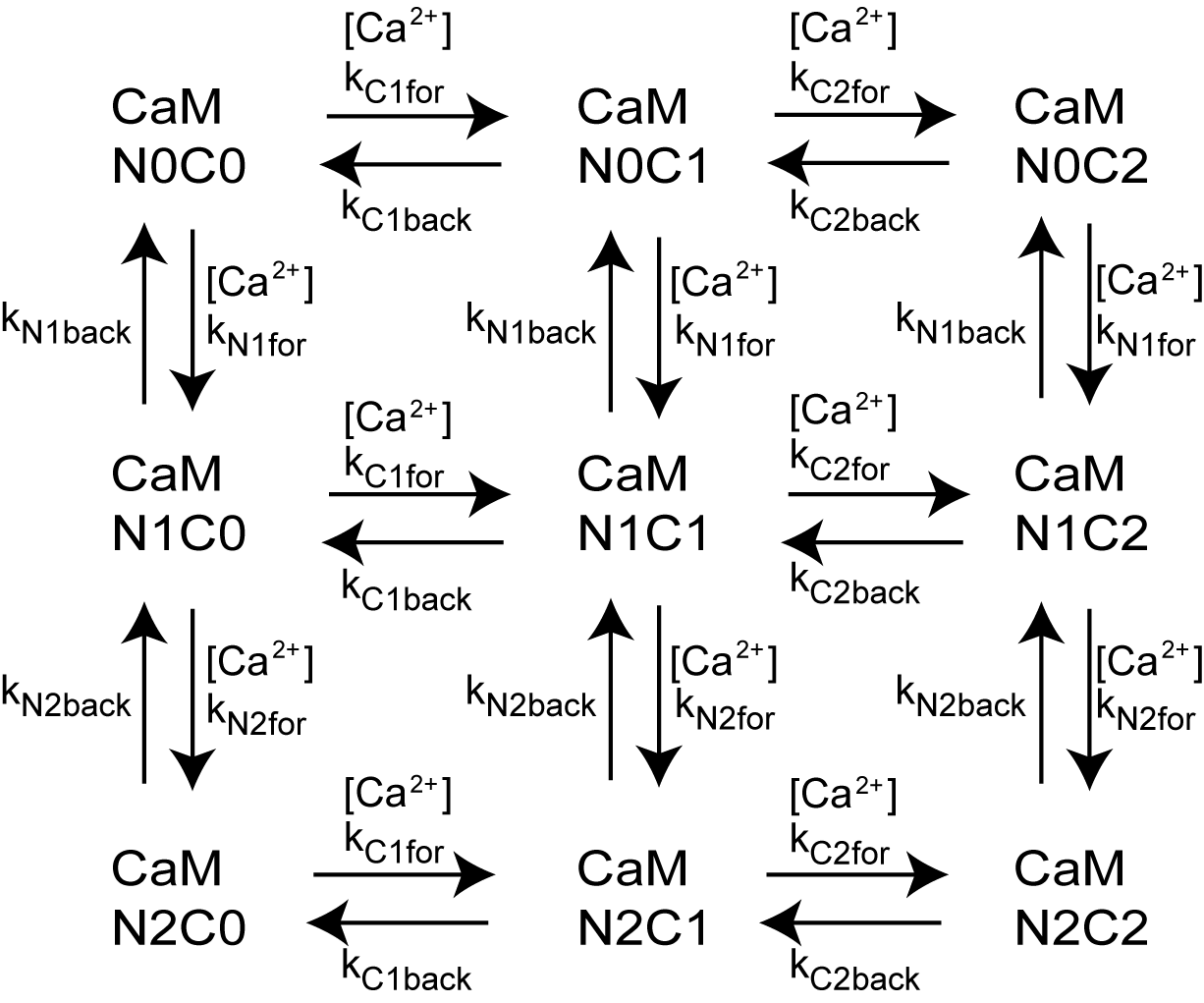

Supplement: Figure S2 — Calmodulin state diagram. Each lobe of calmodulin can bind 2 calcium ions independently of the other lobe. The N lobe has faster kinetics than the C lobe. (0.16 MB TIF) [file pone.0002045.s002.tif]

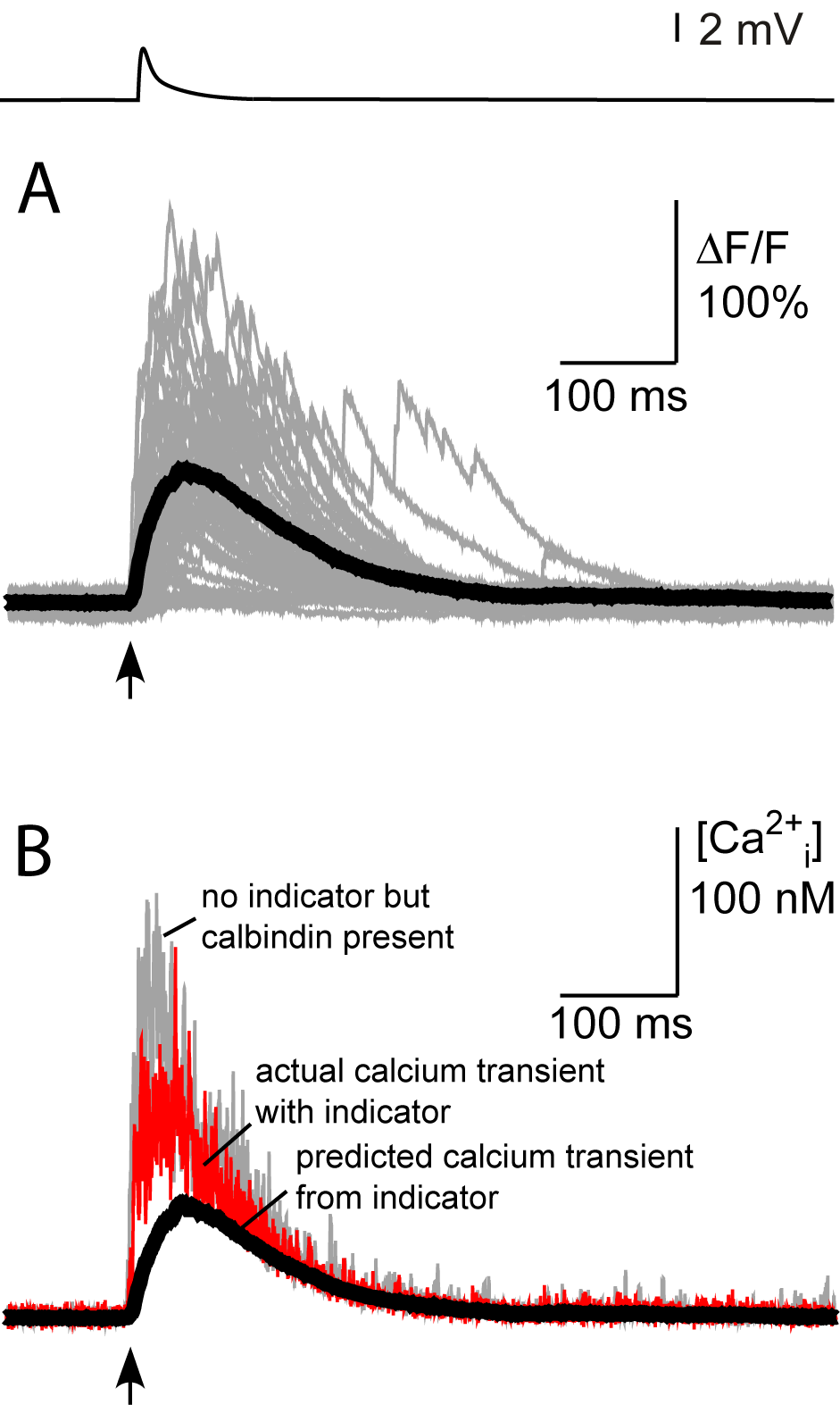

Supplement: Figure S3 — Calcium dynamics following an excitatory postsynaptic potential. (A) Volume-averaged OGB1 fluorescent transients measured in the spine. Thin grey traces are single trials and thick black traces represent the average of 20 trials. (B) Calcium concentration in the spine. The grey trace shows results with 100 µM of OGB1. Also shown is the [Ca2+]pred derived from the fluorescent transient (red). A simulation conducted without OGB1 (black trace) also included 45 µM calbindin-D28k, as would be present in unperturbed neurons. Excitatory postsynaptic potentials were elicited by release of glutamate at times indicated by the arrows. (0.23 MB TIF) [file pone.0002045.s003.tif]

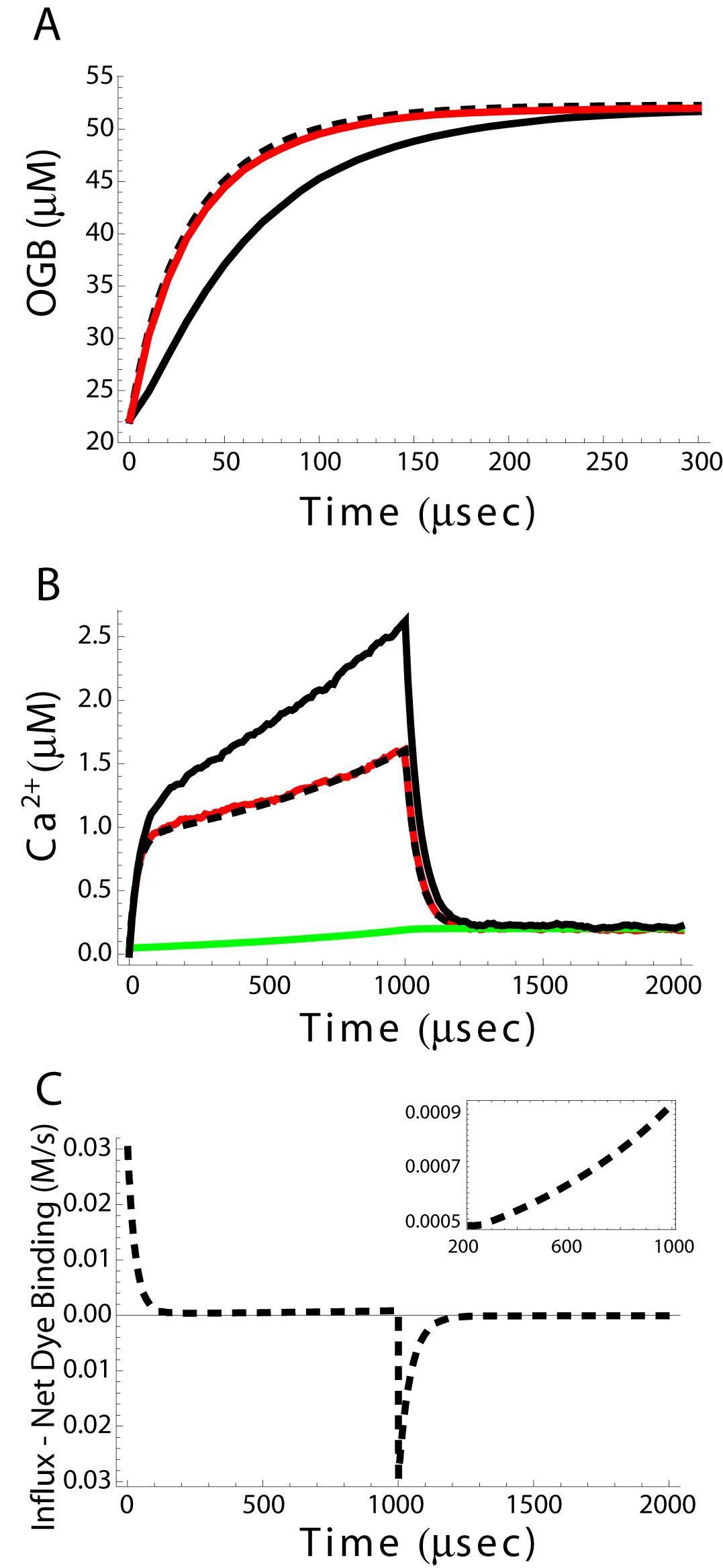

Supplement: Figure S4 — Simulation of 100 µM OGB-1. (A) The predicted (dashed black) and MCell (red) bound OGB-1 closely matched if calcium was uniformly released. If calcium was released from the center of one of the faces in the MCell simulation, it took longer for the system to reach equilibrium (solid black). (B) For a 1 ms duration injection pulse modeled using differential equations, the actual calcium (dashed black) exceeded the indicator-predicted calcium (green). After the pulse was shut off the system quickly equilibrated. If calcium was injected uniformly (red) in an MCell simulation, the results agreed with the differential equation model. However, if calcium was released from the center of one of the faces (solid black) the divergence from the well-mixed unicompartmental model was even greater. (C) The difference between the injected calcium rate and the net rate of binding to the indicator. When channels first opened it took time for the net binding rate to the indicator to approach the influx rate, and during this interval the free calcium increased. This surfeit of free calcium was maintained as long as calcium was injected. The inset shows a magnified difference in rates during the injection. When the injection pulse was shut off the indicator quickly equilibrated with the excess free calcium. (0.28 MB TIF) [file pone.0002045.s004.tif]
